# Unexpectedly high prevalence of familial Mediterranean fever in Slovakia

**DOI:** 10.1007/s10238-025-01634-x

**Published:** 2025-04-01

**Authors:** M. Jesenak, E. Malicherova Jurkova, A. Bobcakova, K. Hrubiskova, O. Petrovicova, L. Kapustova, R. Kosturiak, T. Dallos, A. Markocsy

**Affiliations:** 1 National Centre for Periodic Fever Syndromes, Department of Pediatrics and Adolescent Medicine, Jessenius Faculty of Medicine, Comenius University in Bratislava, University Hospital in Martin, Martin, Slovakia; 2 National Centre for Periodic Fever Syndromes, Department of Pulmonology and Phthisiology, Jessenius Faculty of Medicine, Comenius University in Bratislava, University Hospital in Martin, Martin, Slovakia; 3 Institute of Clinical Immunology and Medical Genetics, Jessenius Faculty of Medicine, Comenius University in Bratislava, University Hospital in Martin, Martin, Slovakia; 4 Outpatient Office of Clinical Immunology and Allergology, 5Th Department of Internal Medicine, Faculty of Medicine, Comenius University in Bratislava, University Hospital Bratislava, Bratislava, Slovakia; 5 Outpatient Office of Clinical Immunology and Allergology, Nitra, Slovakia; 6 Department of Pediatrics, National Institute of Children’s Diseases, Faculty of Medicine, Comenius University in Bratislava, Bratislava, Slovakia

**Keywords:** Familial Mediterranean fever, *MEFV* gene, Slovakia, Prevalence

## Abstract

Familial Mediterranean fever (FMF) is the most common monogenic periodic fever syndrome among all monogenic autoinflammatory diseases. It is characterised by recurrent, self-limited fever attacks of short duration, polyserositis symptoms, and elevated acute-phase reactants. FMF has the highest prevalence in the Eastern Mediterranean region. The first descriptions of FMF cases in Central Europe date back to 2014. The prevalence in the Central European region was previously estimated at 1:465,500 in the paediatric population, with data on adult prevalence lacking. Patients with FMF who fulfilled Eurofever/PRINTO diagnostic criteria were included in the study. Massive parallel sequencing of clinical exome with evaluation of virtual panel for genes associated with inborn errors of immunity and autoinflammatory conditions was used in the vast majority of the patients. Statistical analysis of clinical and laboratory manifestation was performed. The prevalence of FMF in the Slovak population of the present study was 1:48,224 (1:41,348 in children and adolescents), which is significantly higher than expected. The most common variant in our cohort was M694V, present in 52.0% of alleles. Variant K695R was observed in 29.9% which is higher than reported in the other FMF cohorts. Abdominal pain and fever were the most prevalent clinical symptoms, although up to one-third of patients also experienced atypical symptoms such as tonsillitis or cervical lymphadenopathy. This paper provides the first comprehensive analysis of the Slovak National FMF cohort, including epidemiological data, clinical manifestations, and genetic background. Our data could contribute to the general knowledge about FMF characteristics in the underreported regions.

**Supplementary Information:**

The online version contains supplementary material available at 10.1007/s10238-025-01634-x.

## Introduction

Familial Mediterranean fever (FMF) (OMIM 134610, 249,100) is the most common monogenic periodic fever syndrome among all monogenic autoinflammatory diseases. FMF has a high prevalence in the Eastern Mediterranean region and does not meet the criteria for a rare disease in Armenia and Turkey (prevalence ranging from 1:500 to 1:1000) [1, 2]. In Central Europe, the first cases of FMF were identified only recently [3, 4]. The prevalence in the Central European region has been estimated at 1:465,500 among children and adolescents (0–19 years of age) [5].

FMF is characterised by recurrent, self-limited, short-lasting fever attacks; polyserositis symptoms (including peritonitis, pleuritis, and synovitis); and elevated acute-phase reactants [6]. Disease onset typically occurs before the age of 20 years. A typical attack lasts for at least 12 h and usually resolves within 2 days, although it less commonly persists for 3–4 days. The intervals between attacks are clinically symptom-free [7].

Systemic AA amyloidosis is one of the most severe complications of FMF [8]. The primary factor contributing to the development of amyloidosis is the increased production of insoluble serum amyloid A (SAA) secondary to persistent subclinical inflammation. Produced by the liver, SAA can accumulate in the extracellular space of various organs, including the kidneys, liver, spleen, testicles, adrenal glands, gastrointestinal tract, and nervous system, thereby impairing their function.

The treatment of FMF has two main goals: to prevent acute attacks and to suppress chronic subclinical inflammation that could lead to AA amyloidosis. Inflammation in patients with FMF is effectively controlled with colchicine or with treatments targeting interleukin (IL)-1β, which include anakinra (a recombinant human IL-1 receptor antagonist), canakinumab (a human IgG1 monoclonal antibody directed against IL-1β) [9], and rilonacept (a fully human dimeric fusion protein; nonregistered indication) [10]. Colchicine reduces the frequency of attacks, improves quality of life, and most importantly, controls subclinical inflammation, thereby reliably preventing AA amyloidosis. Among patients treated with colchicine, one-third experience partial remission, approximately 5% are nonresponders, and another 2% to 5% cannot tolerate the drug [10].

### Pathophysiology and genetics

FMF is caused by pathogenic mutations in *MEFV* gene that lead to dysfunctional pyrin protein that fails to properly regulate NLRP3 inflammasome activation, leading to uncontrolled IL-1β production [11–15]. Pyrin consists of an N-terminal pyrin domain, a B-box, a bZIP basic region, a coiled-coil domain, and a B30.2 (PRY-SPRY) domain.

Common pathogenic variants (M694V, M680I, V726A, and M694I) are located in exon 10 which is considered the most critical in FMF pathophysiology, encodes a part of the B30.2 domain [16, 17]. Homozygosity for the M694V variant has been associated with particularly severe disease, early onset, and a higher risk of AA amyloidosis [18]. Different patterns of inheritance have been described in FMF cohorts, with many *MEFV* variants still subject to controversial interpretation. Most *MEFV* variants are linked to autosomal recessive (AR) inheritance. However, some variants associated with autosomal dominant (AD) inheritance (H478Y, T577S, T577A, T577N, and M694del) have been reported in the literature [12, 19]. In many countries, FMF cohorts are dominated by heterozygous carriers of typical recessive variants, despite extensive searches failing to identify a second disease-causing variant [16, 20]. This phenomenon, termed pseudo-AD (PAD) inheritance, may be explained by modifications at the epigenetic levels, influence of environmental factors, somatic mosaicism, or the concomitant presence of variants in other autoinflammatory genes (polygenic inheritance). Further complicating the genetic landscape are polymorphisms and risk alleles not directly implicated in the disease. These have low penetrance and high population frequency, and they are also present in healthy individuals (L110P, E148Q, P369S, R408Q, and I591T) [21]. FMF remains a clinical diagnosis based on established diagnostic criteria, with confirmation of causal variants serving only as supportive evidence. Notably, in 10% to 20% of patients with FMF, no causal or suspicious *MEFV* gene variants can be identified [22].

## Methods

For the last decade, the National Centre for Periodic Fever Syndromes at the University Hospital in Martin, Slovakia, has been actively raising awareness about autoinflammatory disorders, with a particular focus on FMF. This has been achieved through targeted lectures delivered at national medical and scientific meetings, as well as campaigns conducted via media and social networks.

For this study, patients with confirmed or suspected FMF were referred to the National Centre for Periodic Fever Syndromes at the University Hospitals in Martin and Bratislava, Slovakia, by general practitioners, immunologists, rheumatologists, and gastroenterologists for evaluation, treatment, and genetic testing. Most of these patients are monitored at the National Centre, which also maintains the National Registry for Periodic Fevers.

Age at first manifestation, age at diagnosis, diagnostic delay, symptoms, clinical presentation (fever, arthritis, abdominal pain, headache, fatigue, erysipelas-like erythema, tonsillitis, lymphadenopathy, chest pain, pleuritis, pericarditis, peritonitis, aphthous stomatitis, and histologically verified amyloidosis), selected laboratory parameters (SAA during stable periods, IgA, and IgD), therapy (use of colchicine, anakinra, canakinumab), and results of genetic testing were analysed. In the Results section, continuous data not conforming to a normal distribution are presented as median values followed by range (minimal to maximal value). The diagnosis of FMF was established in accordance with international consensus guidelines, based on clinical and laboratory symptoms and using the Eurofever/PRINTO diagnostic criteria (Table 1) [23, 24]. Homozygosity/compound heterozygosity for pathogenic or likely pathogenic variants was considered as confirmatory *MEFV* genotype. Heterozygosity for pathogenic or likely pathogenic variants was considered as not confirmatory *MEFV* genotype. Patients who fulfilled Eurofever/PRINTO clinical only criteria were included even though if they were carriers of variants of uncertain significance (VUS) or with negative genetic test results for the *MEFV* gene and other autoinflammatory genes.

**Table 1:** Eurofever/PRINTO criteria

Eurofever/PRINTO clinical-genetic criteria	Eurofever/PRINTO clinical only criteria
Clinical manifestation:	Presence of:
1. Duration of episodes 1–3 days	1. Eastern Mediterranean ethnicity
2. Arthritis	2. Duration of episodes 1–3 days
3. Chest pain	3. Arthritis
4. Abdominal pain	4. Chest pain
	5. Abdominal pain
	Absence of
	6. Aphthous stomatitis
In presence of confirmatory *MEFV* genotype, at least one positive of above	7. Urticarial rash
- Homozygosity/compound heterozygosity for pathogenic or likely pathogenic variants	8. Maculopapular rash
	9. Painful lymph nodes
In presence of not confirmatory *MEFV* genotype, at least two positive of above	
- Heterozygosity for pathogenic/likely pathogenic variants or biallelic VUS	
	FMF ≥ 6 criteria

### Molecular-genetic analysis

Genomic DNA was extracted from ethylenediaminetetraacetic acid-anticoagulated whole blood. Sequencing of the coding region (exons 1–10) and exon–intron boundaries of *MEFV* (NM_000243.3) was conducted using massive parallel sequencing (MPS). MPS was performed with a minimum depth of coverage of 20x, utilising the Clinical Exome Solution Kit from Sophia Genetics (Illumina NextSeq 550; Illumina, San Diego, CA, USA), with in silico copy number variation analysis. Data were analysed using Sophia DDM analysis software. Variants identified via MPS were confirmed by Sanger sequencing. Primer sequences and polymerase chain reaction conditions are available upon request. If a pathogenic or likely pathogenic variant was identified in a proband, DNA from family members was analysed by direct sequencing of the exon carrying the variant. If only VUS was identified in *MEFV* gene in proband using MPS, other symptomatic family members were tested also by using MPS of clinical exome. The nomenclature of identified variants follows the recommendations of the Human Genomic Variation Society [25]. Coding DNA nucleotide numbering and protein sequence numbering were referenced against GenBank sequences NM_000243.3 and NP_000234.1. Variant interpretation was based on the criteria established by the American College of Medical Genetics and Genomics [26] with the support of the Varsome database, Infevers database, InterVar tool, and UniProt database.

### Statistical analysis

The entire cohort was subdivided into subgroups based on zygosity. Patients with two pathogenic or likely pathogenic variants (in homozygous or compound heterozygous states) were classified as patients with AR FMF. Patients with one pathogenic or likely pathogenic variant were classified as patients with PAD FMF. Patients with one pathogenic or likely pathogenic variant and VUS in trans position were marked as P-LP/VUS. The remaining patients were carriers of VUS in a heterozygous state (marked as VUS) or had negative molecular-genetic testing for autoinflammatory genes and fulfilled Eurofever/PRINTO clinical only criteria (marked as FMF without genetic confirmation). The cohort was further subdivided based on genotype, and the subgroups were compared for the studied parameters. Statistical analysis was performed using jamovi software (The jamovi project, version 2.5). For nominal data, the χ^2^ test was used. For comparisons involving more than two groups of nominal data, statistical differences were determined using the χ^2^ test (r x k) or the Fisher–Freeman–Halton test. Continuous data were tested for normal distribution using the Shapiro–Wilk normality test and a QQ plot. Statistical differences were determined using a parametric independent-samples Student’s t test or a nonparametric Mann–Whitney U test. For comparisons involving more than two groups, statistical differences were determined using a parametric one-way analysis of variance or nonparametric Kruskal–Wallis testing with Dwass, Steel, Critchlow–Fligner pairwise comparison analysis. Differences were considered statistically significant at *p* < 0.05.

## Results

### Demographic data

The Slovak national FMF cohort currently comprises 113 living patients (53 males and 60 females; 26 children [< 19 years of age] and 87 adults). The median age at first manifestation was 8 years (range: 6 months–51 years), and the median age at diagnosis was 34.9 years (range: 21 months–74 years), resulting in a diagnostic delay of 11 years (range: 0–58 years). First clinical manifestations before the age of 20 years were observed in 70 patients (61.9%). Detailed demographic data are presented in Table 2 and Supplementary Table S1.

**Table 2:** Demography of FMF cohort

Demography			
Number of patients		113	
Diagnosis	Median age at first manifestation (range) [years]	8 (0.5–51)	
	Median age by diagnosis (range) [years]	34.9 (1.75–74)	
	Median delay of diagnosis (range) [years]	11 (0—58)	
Clinical and laboratory manifestation	Abdominal pain	104	92.9%
	Fever	97	86.6%
	Fatigue	78	69.6%
	Arthritis	78	69.6%
	Chest pain	52	46.4%
	Elevation of SAA during stable period	48	42.9%
	Lymphadenopathy	39	34.8%
	Tonsillitis	35	31.3%
	Headache	29	25.9%
	Aphthous stomatitis	16	14.3%
	Erysipelas-like erythema	16	14.3%
	Pleuritis	15	13.4%
	Peritonitis	15	13.4%
	Pericarditis	8	7.1%
	Amyloidosis	5	4.5%
	Elevation of IgD	5	4.5%
Therapy	Colchicine	99	87.6%
	Canakinumab	17	15.0%
	Anakinra (on demand)	8	7.1%
	Colchicine intolerance	4	3.6%
	Anakinra (regularly)	1	0.9%

Clinical and laboratory manifestation data were counted on 112 patients (without one patient with FMF type 2). Therapy data were counted on all 113 patients. SAA was sampled at least 2 weeks from the last flare. At least one type of serositis (pericarditis, pleuritis, peritonitis) was observed in 33.9% of patients. Abbreviations: *SAA* serum amyloid A, *SD* standard deviation.

### Ethnic origin

We observed a risk ethnic origin (Armenian, Northern Macedonian, Bosnian, and Italian) in 18 patients (15.9%) (Supplementary Table S1). Patients with a risk ethnic origin more frequently experienced tonsillitis (55.5 vs. 26.6%, *p* = 0.015) and fever (100 vs. 81.9%, *p* = 0.05), but less frequently peritonitis (0.0 vs. 14.9%, without a significant difference, *p* = 0.08) compared with patients of Slovak origin. Significant differences were observed between the two groups in terms of zygosity (*p* = 0.003) (Supplementary Table S2).

### Family history

A positive family history of FMF was observed in 59 patients (52.2%) (Supplementary Table S1). Sporadic patients had a lower age at diagnosis (*p* = 0.003) and a shorter diagnostic delay (*p* = 0.004). Patients with a positive family history less frequently experienced fever (81.7 vs. 97.4%, *p* = 0.021) and serositis (15.0 vs. 42.1%, *p* = 0.003).

### Clinical and laboratory manifestations

Abdominal pain and fever were the most common clinical manifestations, observed in 92.9 and 86.6% of patients, respectively (Table 2). Arthritis and fatigue were reported in 69.6% of patients, while chest pain was present in almost half. At least one type of serositis (pericarditis, pleuritis, or peritonitis) was identified in 33.9% of patients. Tonsillitis and lymphadenopathy were observed in approximately one-third of patients, and erysipelas-like erythema occurred in 14.3%.

Adults had significantly higher frequencies of arthritis (73.9 vs. 50.0%, *p* = 0.021) and chest pain (52.3 vs. 23.1%, *p* = 0.009) than children, but lower frequencies of tonsillitis (20.5 vs. 65.4%, *p* < 0.001) and lymphadenopathy (26.1 vs. 61.5%, *p* < 0.001).

All but four patients exhibited elevated C-reactive protein (CRP) levels during flares (range: 9.3–255, mean: 82.7, median: 40.8 mg/L). The four patients with normal CRP levels during flares showed elevated levels of IL-6, SAA, or serum calprotectin during flares (sampling at the same time as CRP) in comparison with flare-free period. IgD testing was performed in 62 patients (range: 6.6–462.0 mg/L, mean: 30.8, median: 13.4 mg/L, reference range: 7.7–132.1 mg/L). All patients except two (462 and 133 mg/L) had normal IgD levels. Mevalonate kinase deficiency was ruled out through molecular-genetic testing. Elevated SAA levels during stable periods (sampling performed at least 2 weeks after the last flare) were observed in 42.9% of patients.

Histologically proven systemic AA amyloidosis was observed in 5.3% of patients. The kidneys were affected in four patients, while the gastrointestinal tract and liver were affected in one patient. Among patients with amyloidosis, the four observed genotypes were M694V/M694V; M694V; K695R, and M694V/E148Q.

One patient presented with multi-organ amyloidosis involving the kidneys, intestine, and peripheral nervous system. Molecular-genetic testing in this patient was performed using MPS of the clinical exome (clinical exome sequencing) with a virtual panel of genes associated with amyloidosis and inborn errors of immunity including other genes associated with autoinflammation. The testing identified variant K695R in *MEFV* gene, without other pathogenic, likely pathogenic variants, or VUS in genes of interest. This patient lacked periodic fever, consistent with FMF type 2 (amyloidosis only).

### *MEFV* variants

Molecular-genetic testing of the *MEFV* gene identified 11 unique missense variants. The variant M694V was the most common, representing 66 alleles (52.0%). The allelic distribution within the Slovak FMF cohort is detailed in Table 3 and genotypic distribution in Table 4. We identified five different pathogenic variants, two likely pathogenic variants, and four VUS. A full list of the identified *MEFV* gene variants in the Slovak FMF cohort is presented in Table 5 and extended version in Supplementary Table S5. The vast majority of pathogenic and likely pathogenic variants (6 of 7, 85.7%) were located in exon 10, which encodes the B30.2 domain. This domain also accounted for most of identified variants (7 of 11, 63.3%). Almost all variants received a benign prediction score using predictive software tools such as combined annotation-dependent depletion (CADD), sorting intolerant from tolerant (SIFT), functional analysis through hidden Markov models (FATHMM), and MetaRNN. Variants classified as VUS have been widely reported in multiple FMF cohorts (59–268 publications). The variants P369S and R408Q were exclusively observed as part of complex allele ([P369S; R408Q]).

**Table 3:** Allelic distribution in Slovak FMF cohort

Allele	Number	Percentage (%)
M694V	66	52.0
K695R	38	29.9
[P369S; R408Q]	5	3.9
E148Q	4	3.1
V726A	4	3.1
R761H	4	3.1
M694I	2	1.6
A744S	2	1.6
F479L	1	0.8
M680I	1	0.8

The total number of alleles is 127 in 113 patients. Complex alleles are bounded in angle brackets. Complex allele was present in 3,9% of alleles

**Table 4:** Genotypic distribution in Slovak FMF cohort

Genotype	Number of patients	Percentage (%)
AR FMF	19	16.8
PAD FMF	74	65.5
P-LP/VUS	4	3.5
VUS	7	6.2
FMF without genetic confirmation	9	8.0
M694V	44	38.9
K695R	30	26.5
FMF without genetic confirmation	9	8.0
M694V/K695R	6	5.3
M694V/M694V	4	3.5
[P369S; R408Q]	3	2.7
M694V/R761H	3	2.7
A744S	2	1.8
E148Q	2	1.8
K695R/[P369S; R408Q]	2	1.8
M694V/E148Q	2	1.8
M694V/V726A	2	1.8
M680I/V726A	1	0.9
M694I/R761H	1	0.9
M694V/M694I	1	0.9
F479L/V726A	1	0.9

First part of the table represents genotypes based on zygosity; second part represents the distribution of particular genotypes. Complex allele is bounded in angle brackets. Variants in trans position are separated by slash symbol. Total number of patients is 113

**Table 5:** Variants in Slovak population

Exon	Domain of the protein	cDNA numbering (NM_000243.3**）**	Effect on protein (short)	Variant type	Allele frequency	ACMG criteria	Infevers	Evaluation
2		c.442G>C	E148Q	missense	1.31%	benign	VUS	VUS
3		c.1105C>T	P369S	missense	0.74%	VUS	VUS	VUS
4	b-BOX	c.1223G>A	R408Q	missense	0.74%	VUS	VUS	VUS
5		c.1437C>G	F479L	missense	0.003%	likely pathogenic	likely pathogenic	likely pathogenic
10	B30.2	c.2040G>C	M680I	missense	0.005%	pathogenic	pathogenic	pathogenic
10	B30.2	c.2080A>G	M694V	missense	0.015%	pathogenic	pathogenic	pathogenic
10	B30.2	c.2082G>A	M694I	missense	0.005%	pathogenic	pathogenic	pathogenic
10	B30.2	c.2084A>G	K695R	missense	0.37%	likely pathogenic	VUS	likely pathogenic
10	B30.2	c.2177T>C	V726A	missense	0.046%	likely pathogenic	pathogenic	pathogenic
10	B30.2	c.2230G>T	A744S	missense	0.14%	likely bening	VUS	VUS
10	B30.2	c.2282G>A	R761H	missense	0.005%	pathogenic	likely pathogenic	pathogenic

Data were collected using Varsome database, Infevers database

*ACMG* American College of Medical Genetics, *VUS* variant of uncertain significance

### Genotype–phenotype correlation

We studied genotype–phenotype correlations of clinical and laboratory manifestations in patients according to zygosity (Supplementary Table S3) and genotype (Supplementary Table S4). There was no difference in the age at first manifestation (*p* = 0.344) between the groups divided according to zygosity. Homozygotes for M694V had a lower age at first manifestation (8.3 ± 4.0 years) than patients with other genotypes (Supplementary Table S4). We found no significant differences in clinical and laboratory symptoms between groups divided by zygosity (Supplementary Table S3) and genotype (Supplementary Table S4).

We applied Eurofever/PRINTO clinical only criteria (Table 1) on all patients in our cohort and studied the distribution in the subgroups according to zygosity and genotype. Seventeen AR FMF patients (89.5%) fulfilled clinical only criteria—more than 6 points (pts), only two patients, with genotypes M694V/M694I and M694V/K695R, had 5 pts, but fulfilled clinical-genetic criteria (duration, abdominal pain, chest pain).

Heterozygotes for M694V fulfilled clinical only criteria in 79.5% of cases (35 patients). Other nine patients fulfilled clinical-genetic criteria.

Heterozygotes for K695R fulfilled clinical only criteria in 79.3% of cases (23 patients, without one patient with FMF type 2). Other six patients fulfilled clinical-genetic criteria and had positive family history with segregation of K695R variant in affected first-degree-relatives. Both patients with K695R/[P369S; R408Q] had 7 pts.

All patients with heterozygosity for VUS or without suspicious variant in *MEFV* gene fulfilled Eurofever/PRINTO clinical only criteria.

We identified 16 patients without fever (14 with PAD FMF and 2 with monoallelic VUS). Among the pathogenic or likely pathogenic variants, eight patients were heterozygous for M694V, six were heterozygous for K695R, one had [P369S; R408Q], and one was heterozygous for A744S. All patients but one with FMF type 2 presented with typical symptoms such as arthritis, chest pain, or abdominal pain, which responded favourably to colchicine (showing a decrease or elimination of attacks). All patients (except one with FMF type 2) fulfilled Eurofever/PRINTO clinical only criteria.

### Treatment

Colchicine treatment was continuously used by 88.4% of patients. Anti-IL-1 treatment was administered to 26 patients (23.0%) because of either an insufficient response to the maximum tolerated dose of colchicine or intolerance.

Canakinumab, a monoclonal antibody targeting IL-1β, was used in 15.2% of patients (17 patients; from which: four AR FMF, nine PAD FMF, one P-LP/VUS (8 pts), one VUS (7 pts), two patients without genetic confirmation (7 and 8 pts), with a dose of 150 mg every 4 weeks in nine patients and 300 mg every 4 weeks in eight patients.

Anakinra, an IL-1 receptor antagonist, was used on demand during sporadic flares in eight patients (M694V/M694V; M694V; K695R; M694V/K695R; one patient without genetic confirmation with 9 pts), and as a regular daily treatment in one patient (M694V/E148Q; 8 pts) (Table 2). Colchicine intolerance was observed in four patients (3.5%).

## Discussion

To date, the Slovak national cohort comprises 113 living patients (53 males and 60 females) with FMF. The prevalence of FMF in the Slovak Republic, based on this study, is 1:48,224 (total population: 5,449,270, according to the 2021 population census). Among children and adolescents (0–19 years of age), the prevalence is 1:41,348 (child population: 1,075,048, according to the 2021 population census). The prevalence of FMF in the Slovak population is thus higher than expected when compared with published data from other Central and Eastern European countries, despite earlier suggestions that FMF might present more mildly in these regions due to environmental modifiers [5]. Moreover, updated prevalence data are largely missing or not reported for many countries in Central and Eastern Europe.

Historical aspects of migration may help explain the higher prevalence of FMF in Slovakia. Following the victory at the Battle of Mohács in 1526, Ottoman Empire forces, led by Suleiman the Magnificent, raided the Kingdom of Hungary, which included the south-eastern part of present-day Slovakia. This resulted in the annexation of the territory for approximately 150 years, potentially allowing genetic inflow from the Eastern Mediterranean region [27]. Another significant population group is the Ashkenazi Jewish community, whose ancestors from the Rhineland migrated to present-day Slovakia with the forces of the Holy Roman Empire around the ninth to tenth century [28].

### Age at manifestation

Only two-thirds of patients (61.9%) experienced their first manifestation before the age of 20 years, which is lower than the 90% typically reported in the literature [29]. This discrepancy could be attributed to a milder clinical presentation influenced by environmental modifiers in the Central European region. Another factor might be a potential bias in our study, as many patients were diagnosed at an older age, and the age of disease manifestation was often reported retrospectively, with significant time delays. Nevertheless, our findings highlight that FMF should also be considered in patients whose periodic fever symptoms begin later in life. Patients with a risk ethnic origin were more likely to exhibit an AR inheritance pattern than those without a risk ethnic origin (*p* = 0.003) (Supplementary Table S2). The PAD inheritance observed in patients without a risk ethnic origin may be explained by pathogenic variants in other genes associated with autoinflammatory diseases. However, given the methodology used for genetic testing in our study, these possibilities were largely excluded for our cohort. Interestingly, sporadic patients showed a lower age at diagnosis (*p* = 0.003) and shorter diagnostic delay (*p* = 0.004) compared with familial cases. This difference may be due to the segregation analysis of undiagnosed symptomatic first-degree relatives of FMF patients (parents, grandparents) at older ages. This phenomenon could also explain the higher age at diagnosis and longer diagnostic delay observed in the cohort as a whole.

### *MEFV* gene variants

The genotype distribution in our cohort aligns with data from Europe and Turkey [30, 31], showing a predominance of PAD (65.5%) and an AR inheritance pattern in 16.8% of patients with FMF. The most common variant was M694V, present in 52.0% of alleles which is consistent with data from Turkey, where Sari A. et al. identified M694V in 54.9% of alleles [32]. On the other side, the number is higher than in Iran, where Alibakhshi R. et al. identified M694V in 20.3% of alleles [33].

We observed five pathogenic variants (M680I, M694V, M694I, V726A, and R761H). Variant K695R located near the most critical region of the protein (M694) is controversial according to the literature [10, 11], although in more cohorts it was evaluated as likely pathogenic [31–34]. We considered it as likely pathogenic with low penetrance taking into consideration the ACMG criteria, clinical manifestation, family history in our patients, and the presence in already published FMF cohorts [31, 32]. It had high prevalence in our cohort (29.9% of alleles) what is much higher than reported in other FMF cohorts from Turkey and Iran (around 1.0%) [31–33]. Twenty-five cases fulfilled Eurofever/PRINTO clinical-genetic criteria, and importantly also clinical only criteria. Other six cases fulfilled clinical-genetic criteria and these patients had positive family history with segregation of K695R variant in affected first-degree-relatives. Another controversial variant, because of its localisation outside exon 10, is F479L [10, 11]. This variant is evaluated as likely pathogenic according to ACMG criteria, Infevers database, Clinvar users, and literature [32, 34]. In our cohort, variant F479L was bound with pathogenic variant in trans position forming AR FMF in one patient (7 pts).

We observed four VUS in our cohort (E148Q, P369S, R408Q, and A744S). In four patients, VUS was bound with pathogenic/likely pathogenic variant in trans position. These patients fulfilled Eurofever/PRINTO clinical-genetic criteria and clinical only criteria, too. Another seven patients with monoallelic VUS fulfilled Eurofever/PRINTO clinical only criteria.

Evaluation of identified variants using databases and prediction tools yielded inconsistent results (Supplementary Table S5). Well-known pathogenic variants were classified as benign by prediction tools such as SIFT, FATHMM, MetaRNN, and CADD. Conversely, VUS like E148Q and P369S had high CADD scores (around 21), suggesting pathogenicity. These inconsistencies highlight the limitations of prediction tools in evaluating *MEFV* variants due to incomplete penetrance in heterozygous carriers and the potential influence of multifactorial inheritance and environmental factors on FMF manifestation. Variants classified as VUS, though widely reported in FMF cohorts, also show higher allele frequencies in healthy populations. This phenomenon may also be explained by incomplete penetrance, multifactorial inheritance, and environmental effects.

### Clinical and laboratory manifestations

Abdominal pain (92.9%) and fever (86.6%) were the most prevalent symptoms in the clinical manifestations. Various types of serositis were observed in nearly a quarter of patients.

Tonsillitis (observed in 35 patients, 31.3%), lymphadenopathy (primarily cervical in 39 patients, 34.8%), and aphthous stomatitis (in 16 patients, 14.3%) are not typical symptoms of FMF, collectively affecting 52 patients (46.4%). All patients in this sub-cohort fulfilled the Eurofever/PRINTO diagnostic criteria. Based on zygosity, the sub-cohort included nine AR FMF patients, 34 PAD FMF patients, one P-LP/VUS patient, four VUS patients (6–7 pts), 4 patients without genetic confirmation (6–7 pts). Recurrent fever attacks persisted beyond the age of 20 years in 34 patients within this subgroup, while 18 patients were still within the childhood age range. The age at onset was > 5 years in 25 patients. Elevated SAA levels during the stable period were observed in 26 patients, while all patients exhibited normal serum IgD levels. No mevalonic aciduria was detected in the subgroup and molecular-genetic testing revealed no pathogenic or likely pathogenic variants in other genes associated with autoinflammatory disorders. Moreover, 53.8% (28 patients) in this subgroup had positive family history for recurrent fever, with segregation of *MEFV* variants in affected first-degree-relatives. Risk ethnic origin had 23.1% (12 patients) from this subgroup. Atypical symptoms, as noted in approximately one-third of patients with FMF from endemic regions, are consistent with reports in the literature [35].

Patients without fever were mostly PAD FMF carriers who experienced attacks of arthritis, abdominal pain, or chest pain, with a favourable response to colchicine in managing the clinical course of the disease. Two patients from this group developed amyloidosis. This phenomenon has been previously reported in the literature [36, 37]. Our cohort contributes to extending the documented number of patients with FMF presenting without fever.

Four patients exhibited normal CRP levels during attacks. These are PAD FMF patients (M694V, K695R) who fulfilled also Eurofever/PRINTO clinical only criteria. These patients showed elevated levels of other inflammatory markers, including SAA, IL-6, and serum calprotectin. These markers were examined during the attacks and at the same time as CRP. Clinically, these patients predominantly experienced fever, arthralgia, and abdominal pain, with a favourable response to colchicine in managing the disease course.

Histology-proven AA amyloidosis was observed in six patients (5.3%), primarily involving the kidneys. Homozygous carriers of M694V are considered to have a higher risk of developing amyloidosis [38, 39]. However, in our cohort, only one patient with amyloidosis was a homozygous carrier of M694V. The remaining five patients were predominantly heterozygous carriers, with M694V identified in two patients and K695R in three patients.

Five patients exhibited symptoms of periodic fever and serositis, while one patient had FMF type 2 (amyloidosis-only phenotype) with involvement of the kidneys, large intestine, and peripheral nervous system. This patient responded well to colchicine therapy, achieving normalisation of SAA levels and renal parameters (creatinine), a reduction in proteinuria, a decrease in the protein-to-creatinine ratio, and no progression of polyneuropathy. The extensive genetic testing of genes associated with amyloidosis and (auto)inflammatory conditions found only variant K695R in *MEFV* gene. We found no prior reports of patients who had FMF with the K695R variant developing amyloidosis. This variant is considered as likely pathogenic with low penetrance so the molecular-genetic finding should be interpreted only according to clinical manifestation and cannot be used for genetic counselling in terms of prognosis in asymptomatic carriers, or family planning.

### Treatment

Colchicine treatment was successfully utilised in the majority of patients with FMF. According to the literature, up to 5% of patients are considered resistant to or inadequately responsive to colchicine, while others may be unable to tolerate the side effects of effective doses [40]. In our cohort, colchicine intolerance was observed in 3.6% of patients, while 23.0% showed an insufficient response to the maximum tolerated dose. These patients experienced a favourable outcome with anti-IL-1 treatment.

Colchicine demonstrated a favourable effect (reduction or elimination of attacks) in all patients from the subgroup with atypical FMF symptoms, including tonsillitis, lymphadenopathy, and aphthous stomatitis. Among these, two patients with AR FMF (M694V) required a combination of colchicine with anti-IL-1 treatment (canakinumab or anakinra on demand) to achieve adequate disease control.

### Weaknesses and limitations

The limitations of our study include the small sample size in the various subgroups and the potential for a margin of error. We focussed on a subgroup of patients with FMF in our cohort who exhibited one or more atypical symptoms for FMF, including tonsillitis, lymphadenopathy, aphthous stomatitis, afebrile attacks, and low CRP levels. Despite these atypical presentations, all these patients met the Eurofever/PRINTO diagnostic criteria (clinical-genetic and clinical only criteria) and responded favourably to colchicine treatment, with a reduction or elimination of attacks. The extensive genetic testing of genes associated with inflammatory conditions was performed in these patients and no pathogenic or likely pathogenic variants were identified. Recurrent fever attacks persisted beyond the age of 20 years in 34 patients from this subgroup, while 18 patients were still in the childhood age. According to the literature, atypical symptoms are present in approximately one-third of patients with FMF.

## Conclusions

The prevalence of FMF in the Slovak Republic is 1:48,224, significantly higher than expected based on available published data, particularly given the low proportion of patients from high-risk regions in our cohort. One-third of patients experienced their first FMF-compatible symptom manifestation after the age of 20 years. Abdominal pain and fever were the most prevalent clinical symptoms, although up to one-third of patients also experienced atypical symptoms such as tonsillitis or cervical lymphadenopathy. Amyloidosis developed in 5.3% of patients. Genetic testing of the *MEFV* gene revealed PAD inheritance in nearly two-thirds of patients and AR inheritance in 16.8%. Patients with a risk ethnic origin were more likely to exhibit an AR inheritance pattern. The most common variant in our cohort was M694V, present in 52.0% of alleles. Variant K695R was observed in 29.9% of alleles what is much higher than reported in other FMF cohorts from Turkey and Iran.

## Supplementary Information

Below is the link to the electronic supplementary material.

Supplementary file1 (DOCX 20 KB)

Supplementary file2 (XLSX 16 KB)

Supplementary file3 (XLSX 19 KB)

Supplementary file3 (XLSX 39 KB)

## Data Availability

The data that support the findings of this study are available from the corresponding authors (AM, AB) upon request.

## Abbreviations

AD: Autosomal dominant
AR: Autosomal recessive
CADD: Combined annotation-dependent depletion
CRP: C-reactive protein
FATHMM: Functional analysis through hidden Markov models
FMF: Familial Mediterranean fever
IL: Interleukin
MPS: Massive parallel sequencing
PAD: Pseudo-autosomal dominant
pts: Points of Eurofever/PRINTO clinical only criteria
SAA: Serum amyloid A
SIFT: Sorting intolerant from tolerant
VUS: Variant of uncertain significance
