# Supplementary material for: Unexpectedly high prevalence of familial Mediterranean fever in Slovakia
Source: Clin Exp Med. 2025 Apr 1;25(1):101. doi: 10.1007/s10238-025-01634-x (PMC11961489; doi:10.1007/s10238-025-01634-x)
Supplement: Supplementary file 1 — Supplementary file1 (DOCX 20 KB) [file 10238_2025_1634_MOESM1_ESM.docx]

**Supplementary Table S1 Demography of specific subgroups of FMF cohort**

| **Demography** | | |
| --- | --- | --- |
|  | **Males** | **Females** |
| Number of patients | 53 | 60 |
| Proportion | 46.9% | 53,1% |
| Median age at first manifestation (range) [years] | 7.0 (1 – 51) | 10.8 (0.5 – 49) |
| Median age by diagnosis (range) [years] | 31 (1.75 – 74) | 38 (4 – 62) |
| Median delay of diagnosis (range) [years] | 10 (0 – 58) | 12 (0 – 54) |
|  | | |
|  | **Children** | **Adults** |
| Number of patients | 26 | 87 |
| Proportion | 23.0% | 77.0% |
| Median age at first manifestation (range) [years] | 2 (0.5 – 8) | 17 (0.5 – 51) |
| Median age by diagnosis (range) [years] | 7 (1.75 – 14) | 40 (10 – 74) |
| Median delay of diagnosis (range) [years] | 4 (0 – 13) | 20 (0 – 58) |
|  | | |
|  | **Risk ethnic origin** | **No risk ethnic origin** |
| Number of patients | 18 | 95 |
| Proportion | 15.9% | 84.1% |
| Median age at first manifestation (range) [years] | 4 (0.5 – 48) | 8 (0.5 – 51) |
| Median age by diagnosis (range) [years] | 29 (0.5 – 62) | 37 (0.5 – 74) |
| Median delay of diagnosis (range) [years] | 10 (0 – 43) | 11 (0 – 58) |
|  | | |
|  | **Positive family history** | **Sporadic patients** |
| Number of patients | 59 | 54 |
| Proportion | 52.2% | 47.8% |
| Median age at first manifestation (range) [years] | 8 (0.5 – 51) | 7 (0.5 – 48) |
| Median age by diagnosis (range) [years] | 40 (0.5 – 74) | 28 (0.5 – 58) |
| Median delay of diagnosis (range) [years] | 23 (0 – 58) | 9 (0 – 44) |

Total number of patients was 113.

**Supplementary Table S2 Distribution of genotypes in Slovak FMF cohort according to ethnic origin**

| **Genotype in patients with risk ethnic origin** | **Number of patients** | **Percentage** |
| --- | --- | --- |
| AR FMF | 8 | 44.4% |
| PAD FMF | 7 | 38.9% |
| P-LP/VUS | 0 | 0.0% |
| VUS | 0 | 0.0% |
| FMF without genetic confirmation | 3 | 16.7% |
| **Genotype** **in patients without risk ethnic origin** | **Number of patients** | **Percentage** |
| AR FMF | 11 | 11.5% |
| PAD FMF | 67 | 70.5% |
| P-LP/VUS | 4 | 4.2% |
| VUS | 7 | 7.4% |
| FMF without genetic confirmation | 6 | 6.3% |

Eastern Mediterranean ethnic origin is considered risky (we observed patients from Armenia, Macedonia, and Italy). Variants in trans position are separated by slash symbol. Abbreviations: *AR FMF* patients with 2 pathogenic or likely pathogenic variants (in homozygous/compound heterozygous state), *P-LP/VUS* patients with one pathogenic or likely pathogenic variant and variant of uncertain significance in trans position, *VUS* carriers of variant of uncertain significance in a heterozygous state
